# Potentiometric Electronic Tongue for the Evaluation of Multiple-Unit Pellet Sprinkle Formulations of Rosuvastatin Calcium

**DOI:** 10.3390/ma17205016

**Published:** 2024-10-14

**Authors:** Patrycja Ciosek-Skibińska, Krzysztof Cal, Daniel Zakowiecki, Joanna Lenik

**Affiliations:** 1 Faculty of Chemistry, Warsaw University of Technology, Noakowskiego 3, 00-664 Warsaw, Poland; pciosek@ch.pw.edu.pl; 2 Department of Pharmaceutical Technology, Faculty of Farmacy, Medical University of Gdansk, Al. Gen. J. Hallera 107, 80-416 Gdansk, Poland; kcal@wp.pl; 3 Chemische Fabrik Budenheim KG, Rheinstrasse 27, 55257 Budenheim, Germany; daniel.zakowiecki@budenheim.com; 4 Department of Analytical Chemistry, Institute of Chemical Sciences, Faculty of Chemistry, Maria Curie-Skłodowska University, M. Curie-Skłodowska Sq. 3, 20-031 Lublin, Poland

**Keywords:** rosuvastatin, electronic tongue, ion-selective electrodes, solid contact

## Abstract

Sprinkle formulations represent an interesting genre of medicinal products. A frequent problem, however, is the need to mask the unpleasant taste of these drug substances. In the present work, we propose the use of a novel sensor array based on solid-state ion-selective electrodes to evaluate the taste-masking efficiency of rosuvastatin (ROS) sprinkle formulations. Eight Multiple Unit Pellet Systems (MUPSs) were analyzed at two different doses (API_50) and (API_10), as well as pure Active Pharmaceutical Ingredient (API) as a bitter standard. Calcium phosphate-based starter pellets were coated with the mixture containing rosuvastatin. Some of them were additionally coated with hydroxypropyl methylcellulose, which was intended to separate the bitter substance and prevent it from coming into contact with the taste buds. The sensor array consisted of 16 prepared sensors with a polymer membrane that had a different selectivity towards rosuvastatin calcium. The main analytical parameters (sensitivity, selectivity, response time, pH dependence of potential, drift of potential, lifetime) of the constructed ion-selective electrodes sensitive for rosuvastatin were determined. The signals from the sensors array recorded during the experiments were processed using Principal Component Analysis (PCA). The results obtained, i.e., the chemical images of the pharmaceutical samples, indicated that the electronic tongue composed of the developed solid-state electrodes provided respective attributes as sensor signals, enabling both of various kinds of ROS pellets to be distinguished and their similarity to ROS bitterness standards to be tested.

## 1. Introduction

Rosuvastatin (ROS) was introduced into medicine in 2003 by Astra Zeneca as *Crestor* and belongs to the statin group of drugs, helping to normalize the concentration of lipids in the blood. As a result of inhibiting the enzyme involved in the synthesis of cholesterol (3-hydroxy-3-methyl-glutaryl-coenzyme A reductase, (HMG-CoA)), they reduce the concentration of “bad” cholesterol (LDL fraction) and triglycerides in the blood and increase the concentration of “good” cholesterol (HDL) in the blood. Therefore, rosuvastatin is used to treat lipid disorders such as too much cholesterol (hypercholesterolemia) or too many triglycerides in the blood. Compared to the most commonly used statins, simvastatin and atorvastatin, it lowers the concentration of LDL cholesterol and triglycerides more strongly.

**Figure 1:**
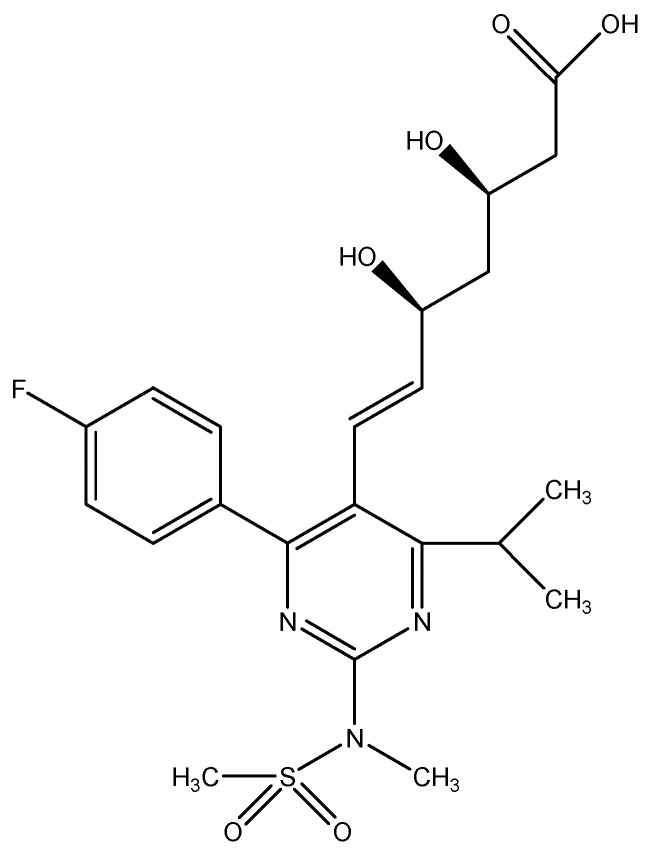
Rosuvastatin((3R,5S,6E)-7-[4-(4-fluorophenyl)-2-(N-ethylmethanesulfonamido)-6-(propan-2-yl)pyrimidin-5-yl]-3,5-dihydroxyhept-6-ene acid).

Rosuvastatin (Figure 1) is a white amorphous powder, which is slightly soluble in water (around 1 mg/mL) and methanol and slightly soluble in ethanol. An additional stable polar methanesulfonamide group in the ROS structure gives it a relatively low lipophilicity compared to other statins, e.g., atorvastatin, fluvastatin, simvastatin, and cerivastatin. As a result, such a hydrophilic compound has limited access to nonhepatic cells because of its low capacity for passive diffusion, and could be avidly taken up into liver cells via selective organic anion transport processes. Due to the hydrophilicity of the molecule and low hepatic metabolism, there is a chance of a lower risk of myotoxicity and adverse drug interactions during rosuvastatin therapy [1,2].

Statins, despite their well-established position in cardiology, are still the subject of research in many research centers around the world. One of the key efforts is the systematic syntheses of new compounds to obtain a substance with the most favorable therapeutic properties, characterized by significant activity against HMG-CoA reductase, and at the same time hepatoselectivity, which will limit side effects [3]. Statins are also used by a large number of manufacturers who produce a variety of dosage forms, including fixed-dose combinations (FDCs). However, statins are known to be chemically unstable. Therefore, many research and scientific centers are working on the development of new pharmaceutical dosage forms, e.g., sprinkle formulations, nanoparticles, or others showing enhanced chemical stability [4,5,6,7].

Like many medicines, rosuvastatin has an unpleasant, bitter taste. Taste masking usually involves preventing a bitter substance from contacting the taste buds. This is achieved by placing such a substance in hard capsules, coated tablets, etc. In the case of sprinkles, the problem is greater, because this form is exposed to the water environment for a longer period of time. Therefore, forms such as sprinkles require special attention. One option is to add substances that change the taste, e.g., sweeteners; however, an appropriate coating with a polymer may be even more effective. Hydroxypropyl methylcellulose (HPMC) is used for this purpose. It is a polymer that is very widely used both in the technology of drug forms and dietary supplements. One of the aims of the study was to compare the bitter taste sensation of rosuvastatin in the presence of isomalt as a sweetener to a formulation additionally coated with HPMC as a bitter substance barrier for the taste buds.

A review of the scientific literature shows that the leading group of research methods for the determination of rosuvastatin, in both biological fluids and pharmaceuticals, are chromatographic methods (high performance liquid chromatography (HPLC) coupled to ultraviolet (UV) detection [8,9] and tandem mass spectrometry (LC–MS/MS) [8]), followed by spectrophotometric UV-VIS methods [8,10,11]. Among electrochemical techniques, potentiometry could be involved, as suggested by the various ion-selective electrodes developed for the determination of similar compounds, such as Atorvastatin [12,13], Fluvastatin [14] and Simvastatin [15]. However, according to our knowledge, there is not any previously published method for testing pharmaceutical samples of rosuvastatin using potentiometry in the literature. Potentiometric methods are simpler, faster and, above all, cheaper, and simultaneously more accurate and precise compared to the above-mentioned methods. These advantages make them very popular and versatile for use in pharmaceutical analysis [16,17,18,19,20].

In addition to the accurate and precise quantitative analysis of pharmaceutical compounds in pharmaceutical products, they can be used in a modern device for automatic analysis and distinction between samples, e.g., drug samples in an electronic tongue (ET).

This is the second area of research related to using ion-selective electrodes and relates to the purpose of this article.

An electronic tongue consists of a so-called sensor array, which is usually composed of electrochemical sensors and a pattern recognition system. Such an array can comprise several to a dozen different electrodes. A potentiometric measurement is often carried out by measuring the electromotive force of a cell consisting of indicator electrodes (which may be different ion-selective electrodes) and a reference electrode under zero-current conditions. The electromotive force (EMF) of the cell depends on the membrane potential of the ion-selective electrode, assuming a constant reference electrode potential under the given measuring conditions. The potential occurs as a result of an electrochemical equilibrium or ionic equilibrium establishing itself at the electrode/solution interface. The potential is described by the Nernst equation and is a function of the concentration (activity) of the ion in the sample solution.

Potentiometric sensors based on polymeric membranes are often used in such arrays due to their undeniable advantages such as, in addition to those mentioned above, a tunable selectivity, proper sensitivity, and a fast response time. Suitably prepared potentiometric sensor arrays can be used to observe the release of both the active substance and excipients to study the masking of bitter taste of drugs [21,22,23,24,25]. As each sensor/ion-selective electrode is broadly selective, i.e., interacts with many constituents of the sample, both with excipients and API, the affinity of each type of electrode in the sensor array towards various types of these constituents is differentiated, giving a characteristic fingerprint to the studied sample [26,27,28].

Therefore, in the present work, we propose a novel sensor array based on solid-state ion-selective electrodes characterized by a tunable sensitivity toward rosuvastatin calcium. For this, the modification of the membrane composition was studied and various plasticizers, lipophilic salts and new ionophores were applies. The main analytical parameters (sensitivity, selectivity, response time, pH dependence of potential, drift of potential, lifetime) of the constructed ion-selective electrodes that were sensitive towards rosuvastatin were determined. Then, the developed electrodes were employed in the sensor array of an electronic tongue applied to the analysis of 10 types of pharmaceutical samples containing rosuvastatin calcium: eight types of ROS pellets and two bitterness standards. The ability of this instrument to discriminate between formulations was assessed by the verification of whether the considered formulations could be discerned with the use of the developed sensor array.

## 2. Materials and Methods

### 2.1. Reagents and Pharmaceutical Samples

The following reagents were used for the preparation of pharmaceutical samples: rosuvastatin calcium (Pharmacin B.V., Zwijndrecht, The Netherlands); PharSQ^®^ Spheres CM calcium phosphate-based starter pellets (Budenheim KG, Budenheim, Germany); microcrystalline cellulose-based pellets (MCC) VIVAPUR^®^ MCC Spheres 710–850 µm (JRS Pharma, Rosenberg, Germany); hydroxypropyl methylcellulose (HPMC)—Tylopur^®^ 606 (ShinEtsu SE Tylose GmbH & Co. KG, Wiesbaden, Gemany); isomalt (Modecor Italiana s.r.l., Cuvio, Italy); talc—Micro ACE P-3 (Nippon Talc Co., Ltd., Osaka, Japan); and propylene glycol (Avantor, Gliwice, Poland).

The following components were applied for the preparation of the polymer membrane: plasticizers: bis (2-ethylheksyl) sebacate (DOS) (Merck Schuchardt OHG, Darmstadt, Germany), diisobutyl phthalate (DIBP) (Fluka, St. Gallen, Switzerland), 2-nitrophenyloctyl ether (NPOE) (Fluka, St. Gallen, Switzerland), polymer: emulsion PVC vinyl chloride (Tarwinyl Tarnów, Poland), lipophilic salts: potassium tetrakis (p-chlorophenyl) borate (KTpCPB) (Fluka, St. Gallen, Switzerland), tetradodecylammonium chloride (TDAC), tridodecylmethylammonium chloride (TDMAC), tributylhexadecylphosphonium bromide TBHDPB (Sigma-Aldrich St. Luis, MO, USA) tetradodecylammonium tetrakis (4-chlorophenyl) borate (TDMA-TCPB) and ionophores: carbonate—ionophore I (ETH 6010, heptyl 4-trifluoroacetyl-benzoate) (Sigma-Aldrich St. Luis, MO, USA), ammonium–ionophore I (nonactin), calcium-ionophore I (ETH 1001, diethyl N,N′-[(4R,5R)-4,5-dimethyl-1,8-dioxo-3,6-dioxaoctamethylene]bis(12-methylaminododecanoate) (Sigma-Aldrich St. Luis, MO, USA) were used. The other reagents: rosuvastatin calcium EP (RSC) from Cadchem Laboratories Limited (Chandigarh, India), Tetrahydrofuran (THF) (POCh, Gliwice, Poland). The sodium salts which acted as interferent anions and other interfering substances were obtained from Fluka, St. Gallen, Switzerland. All chemicals were of analytical-reagent grade. All aqueous solutions were prepared with deionized water of conductivity 0.07 μs/cm (Elix Advantage System Mili-Q plus Milipore, Spittal an der Drau, Austria).

### 2.2. Preparation of the Pellets

Starter pellets were coated using the fluid bed method in the Uni-Glatt apparatus under the conditions described previously. The pellets were coated in two ways:

- Coating of the starter pellets with a single layer containing ROS. In three cases, this layer also contained the sweetener: isomalt (Coating I);
- Coating of starter pellets with two layers, the first containing a drug substance (without sweetener), and the second using an HPMC-based layer (Coating II).

MCC-based pellets were only coated with ROS and sweetener and used as a comparative sample for phosphate-based pellets. Formulations G and H are the same and were used to check the repeatability of the coating process and taste analyses. Additionally, how the size of phosphate pellets affects the taste sensation recorded by electronic tongue was checked. The composition of the coating mixtures and the type of pellets used are shown in Table 1.

### 2.3. Electrode Construction and Membrane Preparation

**Figure 2:**
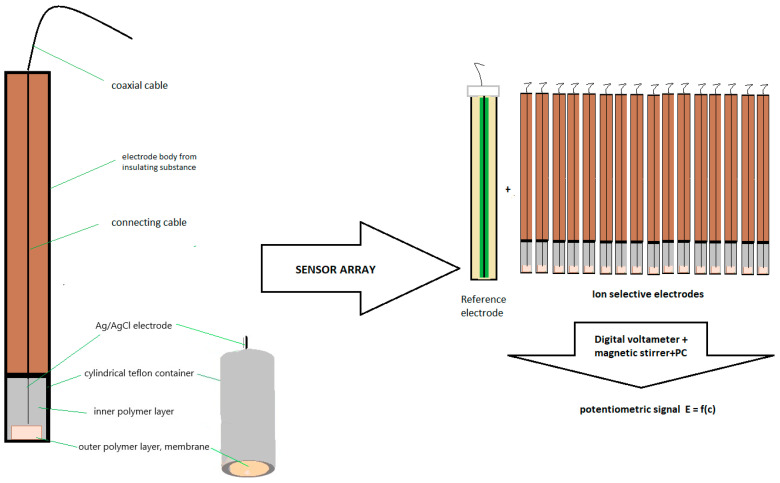
Schematic presentation of ISE and potentiometric sensor array.

The design of constructed electrode was previously reported in other publications [29] and is presented in Figure 2. The membrane of the electrode is 5 mm in diameter and consists of two layers placed in a Teflon holder. The inner layer containing plasticized PVC is in direct contact with the Ag/AgCl electrode and the outer layer composed of plasticized PVC with electroactive substances is in contact with the sample solution. The preparation of the inner layer membrane consists of (1) weighing the layer components, (2) mixing and de-aerating the obtained mixture, (3) filling the Teflon sensor with the mixture, and (4) gelating the layer at a temperature of 353 K for 30 min. The preparation of the outer layer membrane consists of (1) weighing the outer layer components (Table 2), (2) dissolving the obtained mixture in THF, (3) placing it in drops on the inner layer several times, and (4) gelating the layer as a result of THF evaporation at the temperature of 293 K. After the gelation of the two layers, the resulting membranes are in homogeneous polymeric state. The potential in the boundary phase between the inner layer and outer layer is constant and was analyzed in our previous work [30]; it is stable and reproducible. Before the first measurement, the electrodes were preconditioned in 10^−2^ mol L^−1^ NaCl (electrodes no. 1–6, 10–12) and in 10^−3^ mol L^−1^ CaCl_2_ (electrode no. 7), in 10^−3^ mol L^−1^ NH_4_Cl (electrode no. 8), or in 10^−2^ mol L^−1^ Na_2_HPO_4_ + 10^−3^ mol L^−1^ NaCl (electrode no. 9) for 24 h. The electrodes were stored in air between the measurements.

### 2.4. Potentiometric Measurements

Measurements of the electromotive force in the system of ion-selective electrodes (ISE-s) and the reference electrode (Orion 90-02) were carried out at 22 ± 1 °C using a 16 channel Electrochemistry EMF Interface system (Lawson Labs. Inc., Malvern, PA, USA) and IBM PC computer (HP Inc. Hawlett Packard Company China). A Thermo Orion 81-72 glass electrode using a Multifunctional Computer device CX-721 Elmetron (±0.1 mV) (Zabrze, Mikulczyce, Poland) was used for pH measurements.

### 2.5. Electronic Tongue Measurements and Data Analysis

The measurement procedure for the sensor array (Figure 2) was performed to release and test 8 various pharmaceutical samples in the form of pellets and pure API (ROS) at two concentration levels simulating low and high bitter taste. Stabilization of the sensors’ signals was achieved after 5 min of immersion in water (50 mL). Then, adequate pharmaceutical formulations were added into the medium and the resulting signal was observed in terms of the change in potential (EMF) over time. The signals from the sensors were registered for 15 min with 3–5 independent replicates of each formulation. Between sample measurements, the sensors were washed with water and dried.

### 2.6. Electrode Calibration

The electrodes were calibrated by immersing them in a conjugation with a reference Ag/AgCl electrode in a 50 mL aliquots solution of rosuvastatin calcium salt covering the concentration range from 2 × 10^−6^–2 × 10^−3^ mol L^−1^. Potential readings were recorded and plotted against drug concentration in a logarithmic scale. During the measurements the solutions were stirred with a magnetic stirrer and the potential was recorded after stabilization (±0.5 mV).

After the first calibration, among the sensors with a generic anion sensitivity with a near Nernstian slope of the characteristic, selected electrodes (no. 5, 6, 10–12) were reconditioned in ROS solution for 48 h. The remaining sensors, i.e., no. 1–4, were conditioned before calibration in a 10^−2^ mol L^−1^ NaCl; sensors 7, 8, 9 were conditioned in the appropriate solutions as specified in Section 2.3. The electrodes were conditioned in such solutions for 15 min before each next calibration.

## 3. Results and Discussion

### 3.1. Performance Characteristic of Electrodes

In addition to voltammetric, optical sensors, and biosensors, other potentiometric sensors are increasingly used in electronic tongue systems due to their inherent advantages, such as their low cost and simple construction and use. The composition of ISE membranes can be easily tuned and optimized to obtain diverse sensitivities towards various pharmaceutical substances, including both API and excipients. Lipophilic salts, plasticizers, and ionophores are constituents of the membranes of ISEs (Table 2). There is a huge variety of such compounds; moreover, both their type and their proportions will influence sensor selectivity and sensitivity (and all other working parameters) [31,32]. These electrodes can be further exploited in electronic tongue sensing. In the ISEs array of an electronic tongue, ISEs with selective membranes (sensitive towards a particular analyte) and ISEs with cross-selective membranes (sensitive towards many analytes) can be applied. This is a strategy applied in our previous work [33,34,35] where some of the sensors in the sensor array were sensitive to API and others, which were cross-selective, provided signals related to all compounds that have ionic character, including excipients.

In order to prepare a potentiometric sensor array, 12 solid-state ion-selective electrodes with different sensitivities were constructed, including cation- and anion-sensitive electrodes and electrodes sensitive to calcium, ammonium, and carbonate ions. The qualitative and quantitative compositions of polymeric membranes no 1–12 based on polyvinyl chloride are given in Table 2. For each membrane composition, two electrodes were prepared. To check the sensors worked correctly, for all electrodes, analytical parameters such as sensitivity (a), standard potential (b), correlation coefficient of the linear regression curve (R^2^) were determined from the calibration curves for the ROS solutions.

To determine the calibration curves, the potential of electrodes 1–12 was measured sequentially in ROS solutions with concentrations of 2 × 10^−6^, 2 × 10^−5^, 2 × 10^−4^, and 2 × 10^−3^ mol L^−1^ in three replicates, i.e., over three days. For each calibration of an individual electrode, a linear regression equation was determined from which the coefficients a, b, and R^2^ were determined (a total of 36 linear regression equations were obtained). The mean values of the three repetitions of the slope of the linear part of the calibration curve of the characteristic, the standard potential, and the mean R^2^, as well as the standard deviations for these values, were calculated. The mean values obtained are shown in Table 3. These values of the electrode potentiometric response parameters were determined for their linear range, which for each electrode was 2 × 10^−5^–2 × 10^−3^ mol L^−1^, except for electrode no. 1. The experimental data presented (Table 3) show that out of twelve electrodes with different selectivity, eight sensors show optimal potentiometric properties (appropriate sensitivity, linearity range), which allow their appropriate use for pharmaceutical analysis.

The optimal sensors of the electronic tongue can be selected, i.e., cation-selective electrodes containing KTpClBP in the membrane-plasticized DIBP and NPOE (sensors no. 2, 3). The larger group are anion-selective electrodes. The TDAC (no. 11) and TBHDPB (no. 6) membrane electrodes show the closest theoretical responses to rosuvastatin of −63.12 mV decade^−1^ and −56.67 mV decade^−1^, respectively. Apart from the visible dependence on the type of ion exchanger, the type of plasticizer used also affects the sensitivity of the electrodes. For example, among membranes containing tridodecylmethylammonium chloride (electrodes no. 10–12), plasticized with three different solvents, the best performance was obtained for the electrode containing NPOE; a lower slope was observed for the electrode with a membrane containing DOS; and the membrane plasticized with DIBP shows sensitivity higher of 10 mV decade ^−1^, than theoretical one.

Following 2 electrodes selected for the ET sensor array include anion-selective electrode (no. 5 TDMAC + NPOE) and an electrode sensitive to carbonate ions (no. 9), which have very similar slope characteristics, lower than the Nernstian (about −45 mV decade^−1^). The cationic KTpClBP + DOS (no. 1), cationic–anionic (no. 4), calcium, and ammonium electrodes (no. 7 and no. 8) are not suitable for testing pharmaceutical samples containing ROS, due to the limited range of linearity (electrode no. 1), and also their low sensitivity to ROS in the tested determination range (electrode no. 4, 7, and 8).

The electrodes (no. 5, 6, 10, 11, 12 which have anionic function showing the favorable parameters characterized by the most closest near Nernstian response (from about −45 to −68 mV decade^−1^) in the linear range of 2 × 10^−5^–2 × 10^−3^ mol L^−1^ were selected for further research aimed to determine following working parameters such as response time, potential drift, selectivity, and pH dependence.

Summarizing the above, eight electrode types were selected:

- sensitive towards the active substance, i.e., electrodes no. 5, 6, 10, 11, 12;
- with generic cation-selectivity, i.e., electrodes no. 2, 3;
- carbonate-sensitive electrode (no. 9)

They were prepared in two replicates (two sensors for each membrane composition), giving a total 16-sensor array, which was applied for further measurements.

### 3.2. Response Time and Potential Drift

**Figure 3:**
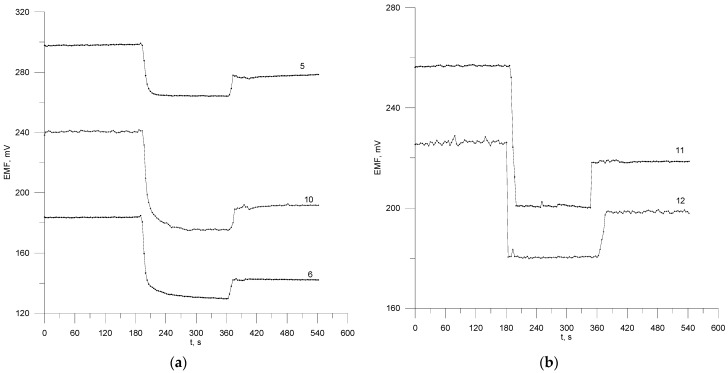
Dynamic response for electrodes no. 5, 6, 10 (**a**) and for electrodes no. 11 and 12 (**b**).

A very important analytical parameter of the electrodes as a function of time is the response time of the sensor to step changes in the analyte concentration and the stability of the potential over time. The response time for the electrodes (Figure 3) was determined using the method of injecting a concentrated ROS solution into the ROS sample being mixed. After the potential became stable, the sample was diluted with deionized water in the ratio of 1 to 1. The response time of the electrodes was determined as the time at which the output signal of the sensor reached 95% of its final value after the equilibrium time in response to a change in the analyte concentration. The values of response time at increasing concentration are 30 s for electrodes no. 5, 6, 10 and 10–15 s for electrodes no. 12, 11. After diluting, these times were relatively shorter and amounted to 10–15 s.

**Figure 4:**
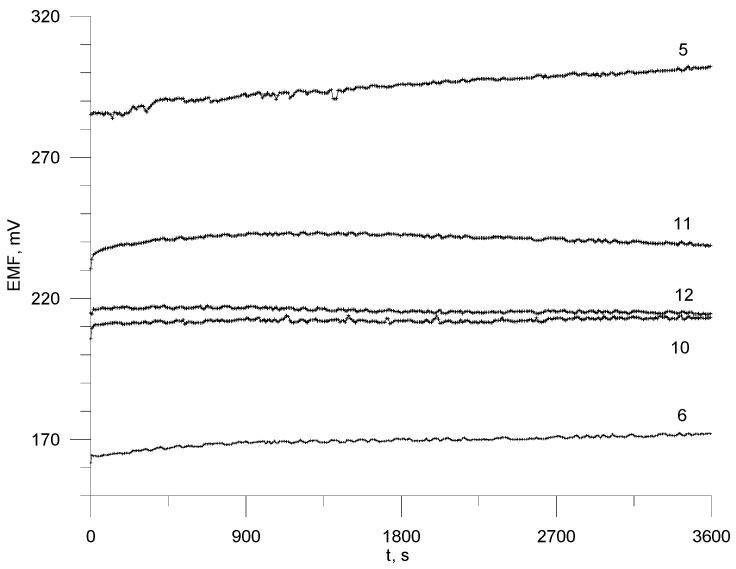
Potential drift of the selected electrodes in 2 × 10^−4^ mol L^−1^ rosuvastatin solution during one hour.

The potential drift o f the developed electrodes was determined by immersing the electrodes in a stirred 2 × 10^−4^ mol L^−1^ ROS solution. The results for electrodes no. 5, 6, 10, 11, 12 are presented in Figure 4. It can be seen that the most stable potential was achieved for sensors no. 10 and 12 with TDAC (4.2–1.5 mV hour^−1^).

### 3.3. Dependence of EMF on pH

The influence of pH on the rosuvastatin electrodes potential was studied in the solution of ROS at a concentration of 2 × 10^−4^ mol L^−1^. The pH was changed using small volumes of HCl (c = 0.5 mol L^−1^) to 50 mL ROS solution. Then, NaOH solution (c = 0.1 mol L^−1^) was added dropwise to the new ROS sample. After each acid or base addition, the solution pH and the electrode potential change were measured.

**Figure 5:**
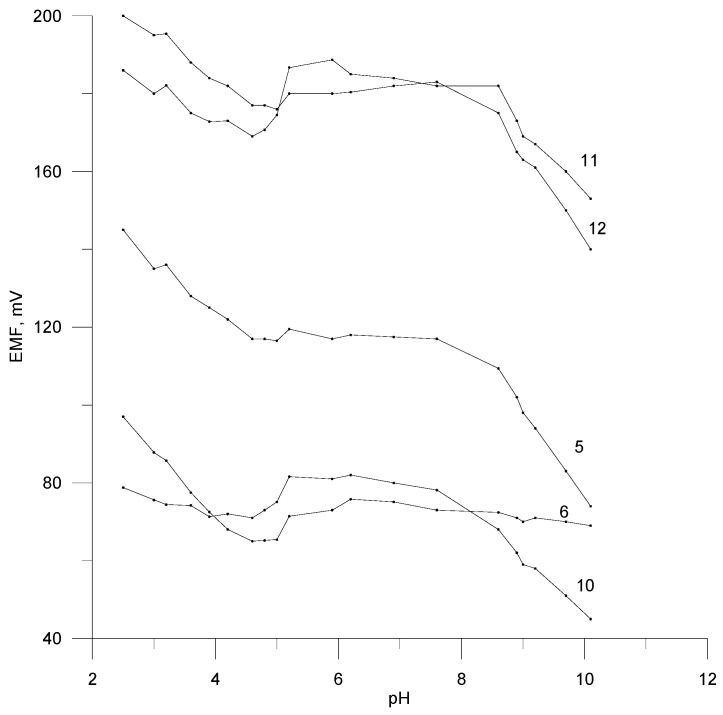
Effect of the pH on the potential response of the selected electrodes in 2 × 10^−4^ mol L^−1^ of rosuvastatin solution.

The effect of pH on the ROS solutions for all electrodes are shown in Figure 5. A stable potential response can be observed for pH ranges of about 5.0–9.0 in the 1 × 10^−4^ mol L^−1^ ROS solution for the majority of electrodes. In the acidic medium, a characteristic jump in potential can be observed. This can be explained by the presence of the acidic form of rosuvastatin in the tested solution.

### 3.4. Selectivity

The effect of some interferents on the potentiometric determination of ROS was evaluated by measuring the selectivity coefficients using the separate solution method (SSM). The potential of a cell comprising a ROS-selective electrode and a reference electrode was measured in the concentration range of 2 × 10^−3^–2 × 10^−6^ mol L^−1^ and 10^−3^–10^−5^ mol L^−1^ for the main and interfering ion solutions, respectively. Selectivity coefficient values were determined at two different concentrations: (1) 10^−3^ mol L^−1^ and (2) 1 mol L^−1^. The following relationship was used to determine the selectivity coefficients using this method (1):

$$logK_{i,j}^{pot}=\left(\frac{E_{J}-E_{i}}{S}\right)-\left(\frac{z_{i}}{z_{J}}-1\right)logc_{i}$$

where J stands for an interfering ion, E_J_ is the potential of the electrode in the interfering ion’s solution, E_i_ is the potential of the electrode in the main ion’s solution, S is the main ion’s slope of characteristics, z_i_ is the charge of the main ion, z_J_ is the charge of interfering ions, c_i_—10^−3^ mol L^−1^.

Determining the values of selectivity coefficients at ion concentrations of 1 mol L^−1^ (2), according to Bakker’s method [36], the following equation was used:

$$K_{i,J}^{pot}=exp\left\{\frac{E_{J}^{0}-E_{i}^{0}}{RT}zF\right\}$$

The results for the selected selectivity coefficients for the studied rosuvastatin ion-selective electrodes with various ion exchangers are presented in Table 4.

It can be observed that all electrodes have similar values of selectivity coefficients in relation to the selected interfering agents. For example. for sensor no. 4., the determined K_i,J_ values are about 10^−6^ (hydrogen phosphate, oxalate, aspartic acid, lactose, urea, tartrate) about 10^−5^ (chloride, sulfate, acetate, citrate, glutamic acid) and the highest values of about 10^−3^ were obtained for anions, i.e., nitrate and benzoate.

### 3.5. Lifetime

**Figure 6:**
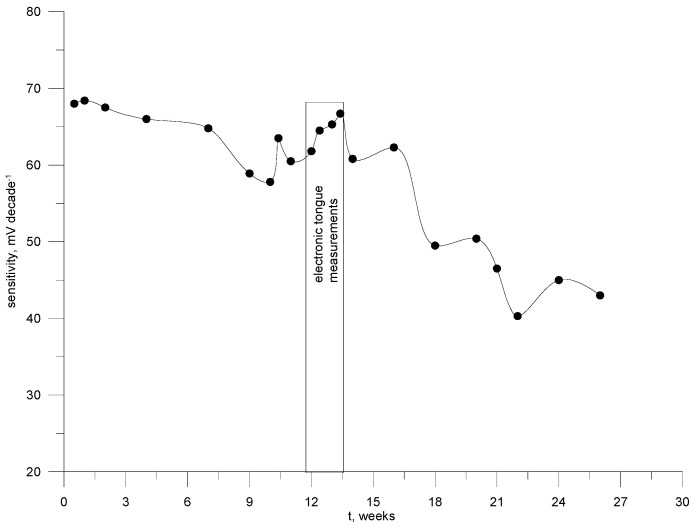
Stability of sensitivity of the electrode no. 11 in time.

The lifetimes were tested by measuring the characteristics slope of the electrodes kept in the air at room temperature. The measurements were made systematically, usually every 14–21 days, in freshly prepared rosuvastatin solutions. The correct working time of the electrodes was determined up to this moment, until they showed a deviation of ± 16% from the Nernstian characteristic slope [37] for electrodes no. 6, 11, 12. and for electrodes no. 5, 10 ± 16% from the determined slope presented in Table 3. The lifetime of these sensors amounted to five months. After this time, the electrode requires only simple regeneration in the second membrane phase and is ready to resume work as if it was a new electrode. Figure 6 shows the time dependence of the sensitivity of electrode no. 11.

### 3.6. Discrimination of Rosuvastatin Calcium Formulations

The sensor array prepared for the electronic tongue consisted of 16 electrodes: no. 2, 3, 5, 6, 9–12. It was applied to check discrimination of the studied ROS formulations suggesting their variable taste masking efficiency. Therefore, apart from the formulations A-H (Table 1), pure APIs at high (API_50) and low doses (API_10) were also considered as bitter standards. Thus, 10 types of samples in total were tested according to the procedure described in the experimental section. The responses of the sensor array recorded five minutes after the start of the release were processed by means of PCA.

**Figure 7:**
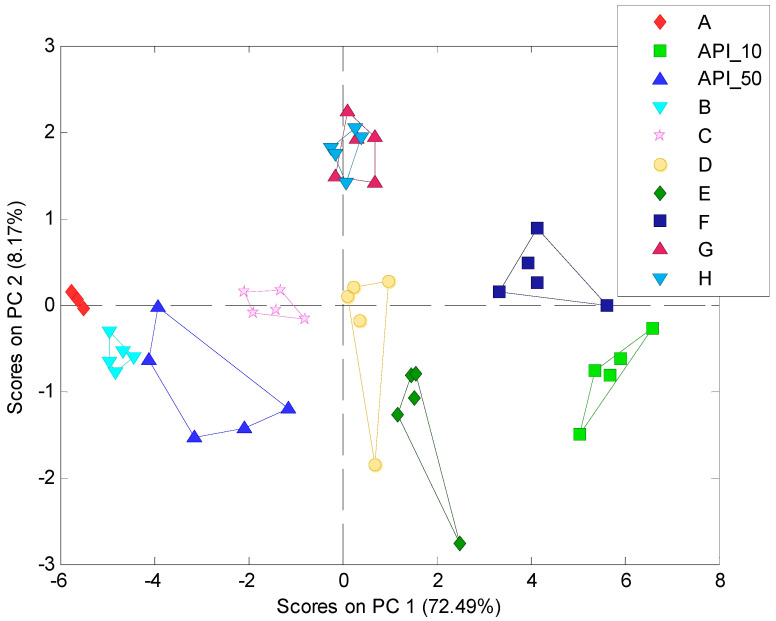
PCA score plot of electronic tongue results for the studied formulations (A–H). and pure API (API_10 and API_50).

Principal Component Analysis (PCA) is a data analysis technique most widely used for electronic tongues data interpretation, mainly for dimensionality reduction and thus visualization of samples (dis)similarities [28]. Its characteristic feature is that the data are linearly transformed onto a new coordinate system and the obtained, new directions, that are called principal components (PCs), capture the largest available variation in the following PCs. The first principal component PC1 will have the largest possible variance, and all consequent principal components will have the largest variance given the constraint that these components are uncorrelated (orthogonal) to the other principal components. In this new PCA space, the samples are represented according to corresponding PCA scores. The highest variability observed in the data is present in the highest PCs; therefore, in most cases in the observation of electronic tongue data, PC1-PC2 subspace is applied, in which samples are represented by PC1 and PC2 scores, respectively. The PCA score plot obtained is presented in Figure 7.

**Figure 8:**
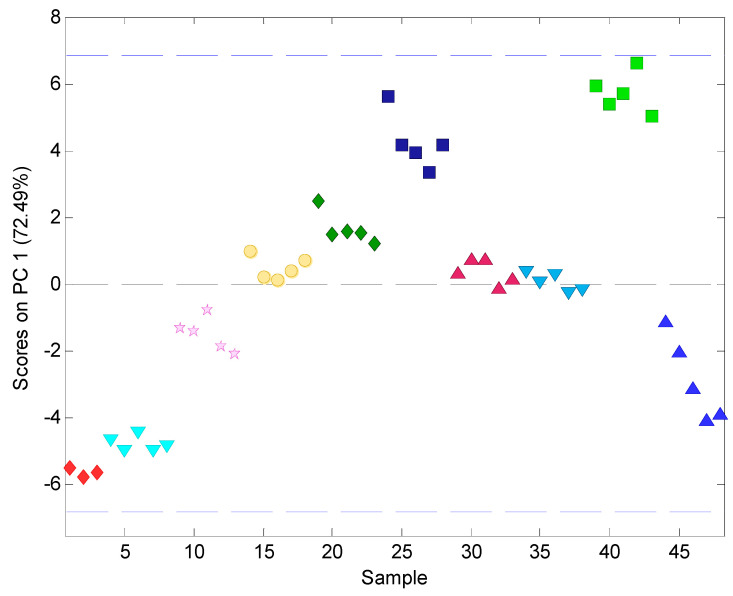
PC1 values of the electronic tongue results showing gradually changing characteristics of the studied formulations (A–H), compared to pure API standards (API_10 and API_50).

Almost all samples formed distinct clusters, that are linearly separable from each other, which proves that the designed electronic tongue exhibits satisfactory discriminatory abilities towards ROS-based pharmaceutical samples. Only in the case of two formulations was an overlap between G and H was observed. On the opposite sides of the plot, along PC1, two bitter standards are visible, whereas most ROS pellet samples are placed in between. Therefore, a higher bitterness (API_50) and lower bitterness (API_10) can be associated with the values of the first Principal Component, which captures most of the variance in the data set (>72%). Most pellet samples show moderate value of PC1, showing moderate characteristics comparable to two studied API standards, which is correct and was expected. To study this effect in more detail, the following samples were presented only as a function of PC1 in Figure 8.

Three main groups can be observed in Figure 8 according to the similarity of their PC1 value:

- The most negative value, comparable to API_50, thus suggesting that the highest bitterness was found for samples A and B;
- Value close to zero associated with moderate bitterness can be noted for samples D, E, G, H;
- The most positive value, compared to API_10, occurred for sample F.

**Figure 9:**
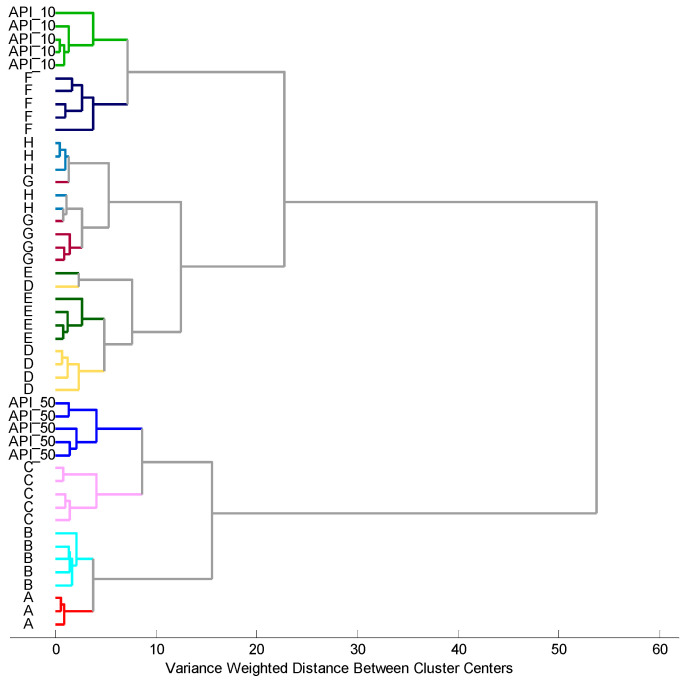
HCA showing the discrimination of ROS samples. Dashed lines represent a division into 2 groups at variance weighted distance > 30, and 3 groups at variance weighted distance ~20.

The grouping of the samples according to their similarity to API_10 and API_50 standards was further studied by Hierarchical Cluster Analysis (HCA) (Figure 9). Clustering is one of the most popular methods among unsupervised machine learning algorithms, enabling us to find structures within the data. The studied samples were partitioned into clusters that share similar attributes according to electronic tongue results. HCA enabled us to visualize a hierarchy of clusters in the form of a dendrogram that has an established ordering from the left to the right side. The shorter branches in the dendrogram linking the specified objects, the higher their similarity.

HCA revealed that the two main groups of samples can be distinguished at a variance-weighted distance > 30: A, B, and C, which are similar to API_50 (higher bitterness); and D. E, F, G, and H, that are similar to API_10 (lower bitterness). Comparing PCA (Figure 7) and HCA many correlations can be observed. Again, F pellets exhibited the highest similarity to API_10, Whereas the A and B pellets to were most similar to API_50. Samples D, E, G, H, presenting moderate characteristics according to the analysis of PC1 (Figure 8), were grouped together with API_50 at a variance-weighted distance > 30; however, they can be regarded as a separate group from the most similar API_10 and F pellets at a variance-weighted distance ~20. Samples G and H, that overlapped on the PCA score plot, are also less distinguishable by HCA. All these findings confirm that the groping of samples visible on the PCA plot (Figure 7), and especially considering the PC1 values (Figure 8), is also reflected on the HCA dendrogram (Figure 9). Thus, the electronic tongue composed of the developed solid-state electrodes provided respective attributes as sensor signals, enabling both various kinds of ROS pellets to be distinguished and their similarity to ROS bitterness standards to be tested.

## 4. Conclusions

The research objective was achieved in this study. It was possible to construct a potentiometric sensor array to evaluate the bitterness of pharmaceutical formulations containing rosuvastatin, which have not yet been investigated. Among the twelve solid contact electrodes, eight sensors were selected, with different membrane compositions that were characterized by different sensitivities, either cationic (electrodes no. 2, 3) or anionic (electrodes no. 5, 6, 9–12). From among the electrodes with anionic function, electrodes with near-Nernst sensitivity were tested. The results of the analytical parameters were favorable, the two sensors containing TDAC and TBHDPB salts in the membrane and NPOE showed the best function and a relatively short response time. The selectivity for all electrodes tested was very good and similar. Taste masking studies of rosuvastatin multiparticulates in sprinkle form were carried out using the sensor array prepared. Using an electronic tongue, it was initially shown that ROS-based pharmaceutical samples could be distinguished (Figure 5). Taste masking compared to the pure API standards was also shown to be most effective for sample F, size XL, medium for samples D and E, size M, and for samples G and H, size XL. The greatest bitterness is shown for samples A and B, size XS, and sample C.

## Figures and Tables

**Table 1:** Composition of mixtures for coating of pellets (g).

	Formulation:	A	B	C	D	E	F	G, H
	Starter Pellets:	PharSQ Spheres CM XS	PharSQ Spheres CM XS	MCC	PharSQ Spheres CM M	PharSQ Spheres CM M	PharSQ Spheres CM XL	PharSQ Spheres CM XL
Coating I:	Rosuvastatin Calcium	5.0	5.0	5.0	5.0	5.0	5.0	5.0
	Tylopur 606	5.0	5.0	5.0	5.0	5.0	5.0	5.0
	Propylene Glycol	0.5	0.5	0.5	0.5	0.5	0.5	0.5
	Talc	0.5	0.5	0.5	0.5	0.5	0.5	0.5
	Isomalt	5.0			5.0			5.0
	Dye	q.s.		q.s.	q.s.			q.s.
	Water	Ad 100.0	Ad 100.0	Ad 100.0	Ad 100.0	Ad 100.0	Ad 100.0	Ad 100.0
Coating II:	Tylopur 606		10.0			10.0	10.0	
	Talc		1.0			1.0	1.0	
	Propylene Glycol		1.0			1.0	1.0	
	Water		Ad 100.0			Ad 100.0	Ad 100.0	

**Table 2:** The composition of membrane phase of prepared ion-selective electrodes.

Lp	Lipophilic Salt (wt.%)	Plasticizer (wt.%)	Ionophore (wt.%)
1	KTpClBP (3%)	DOS (66%)	-
2	KTpClBP (3%)	DIBP (66%)	-
3	KTpClBP (3%)	NPOE (66%)	-
4	CAT-AN (3%)	NPOE (66%)	-
5	TDMAC (3%)	NPOE (66%)	-
6	TBHDPB (3%)	NPOE (66%)	-
7	KTpClBP (0.7%)	NPOE (64%)	Calcium ionophore I (1.3%)
8	KTpClBP (0.7%)	NPOE (64%)	Ammonium ionophore I (1.3%)
9	TDMAC (0.3%)	DOS (62%)	Carbonate ionophore I (0.7%)
10	TDAC (3.5%)	DIBP (64%)	-
11	TDAC (3.5%)	NPOE (64%)	-
12	TDAC (3.5%)	DOS (64%)	-

**Table 3:** Potentiometric characteristic of ion-selective electrodes in rosuvastatin calcium salt solutions with the concentration of 2 × 10^−5^–2 × 10^−3^ mol L^−1^ (mean ± SD, n = 3).

Electrode No. Membrane Composition	a	b	R^2^	Linear Range (mol L^−1^)
1 KTpClBP + DOS	34.36 ± 4.50	136.00 ± 22.35	0.9945 ± 0.0029	2 × 10^−4^–2 × 10^−3^
2 KTpClBP + DIBP	34.70 ± 2.20	167.00 ± 28.45	0.9962 ± 0.,0024	2 × 10^−5^–2 × 10^−3^
3 KTpClBP + NPOE	45.25 ± 0.85	195.00 ± 34.72	0.9949 ± 0.0029	2 × 10^−5^–2 × 10^−3^
4 CAT-AN + NPOE	−14.00 ± 2.50	15,55 ± 35.86	0.9978 ± 0.0020	5 × 10^−5^–2 × 10^−3^
5 TDMAC + NPOE	−45.00 ± 0.50	11.62 ± 22.25	0.9990 ± 0.0009	2 × 10^−5^–2 × 10^−3^
6 TBHDPB + NPOE	−56.67 ± 2.75	−99.17 ± 12.99	0.9961 ± 0.0034	2 × 10^−5^–2 × 10^−3^
7 Ca^2+^ + NPOE	10.50 ± 1.63	130.58 ± 19.89	0.9975 ± 0.0015	2 × 10^−5^–2 × 10^−3^
8 NH_4_^+^ + DOS	5.50 ± 0.50	10.55 ± 25.74	0.9971 ± 0.0020	2 × 10^−5^–2 × 10^−3^
9 CO_3_^2−^ + DOS	−43.05 ± 2.50	45.51 ± 23.84	0.9980 ± 0.0016	2 × 10^−5^–2 × 10^−3^
10 TDAC + DIBP	−68.83 ± 4.04	36.44 ± 45.81	0.9958 ± 0.0029	2 × 10^−5^–2 × 10^−3^
11 TDAC + NPOE	−63.12 ± 4.03	−55.14 ± 18.17	0.9983 ± 0.0014	2 × 10^−5^–2 × 10^−3^
12 TDAC + DOS	−53.58 ± 10.50	212.72 ± 16.60	0.9973 ± 0.0013	2 × 10^−5^–2 × 10^−3^

**Table 4:** Selectivity coefficients of five rosuvastatin ion-selective electrodes using the SSM.

$K_{ROS/interf}^{pot}$										
Electrode No.	5 (TDMAC + NPOE)		6 (TBHDP + DIBP)		10 (TDAC + DIBP)		11 (TDAC + NPOE)		12 (TDAC + DOS)	
C, mol L^−1^	1.0	1 × 10^−3^	1.0	1 × 10^−3^	1.0	1 × 10^−3^	1.0	1 × 10^−3^	1.0	1 × 10^−3^
Cl^−^	2.08 × 10^−5^	5.89 × 10^−3^	6.08 × 10^−6^	1.38 × 10^−2^	1.49 × 10^−5^	6.47 × 10^−3^	3.16 × 10^−5^	8.46 × 10^−3^	4.04 × 10^−6^	7.72 × 10^−3^
NO_3_^−^	3.38 × 10^−3^	6.21 × 10^−2^	3.33 × 10^−3^	1.57 × 10^−1^	2.95 × 10^−3^	1.05 × 10^−1^	2.16 × 10^−3^	1.37 × 10^−1^	2.35 × 10^−3^	1.11 × 10^−1^
SO_4_^2−^	8.59 × 10^−6^	7.37 × 10^−4^	2.71 × 10^−5^	6.71 × 10^−4^	2.08 × 10^−5^	7.67 × 10^−4^	1.24 × 10^−5^	5.07 × 10^−4^	1.73 × 10^−5^	5.62 × 10^−4^
H_2_PO_4_^−^	1.93 × 10^−7^	1.31 × 10^−2^	1.16 × 10^−5^	1.57 × 10^−2^	9.28 × 10^−5^	2.14 × 10^−2^	7.62 × 10^−6^	1.03 × 10^−2^	1.00 × 10^−5^	3.40 × 10^−2^
Acetate	2.66 × 10^−5^	5.01 × 10^−2^	1.78 × 10^−6^	8.44 × 10^−3^	2.20 × 10^−6^	9.78 × 10^−3^	3.27 × 10^−6^	1.17 × 10^−2^	7.91 × 10^−7^	5.98 × 10^−3^
Benzoate	4.95 × 10^−4^	6.35 × 10^−2^	8.16 × 10^−4^	2.93 × 10^−2^	4.02 × 10^−4^	3.65 × 10^−2^	1.16 × 10^−3^	2.27 × 10^−2^	2.38 × 10^−3^	1.93 × 10^−2^
Oxalate	8.45 × 10^−6^	5.60 × 10^−4^	7.17 × 10^−6^	2.00 × 10^−4^	1.67 × 10^−5^	2.29 × 10^−4^	8.67 × 10^−6^	1.31 × 10^−4^	1.46 × 10^−5^	9.90 × 10^−5^
Citrate	3.70 × 10^−6^	7.69 × 10^−5^	6.92 × 10^−6^	4.32 × 10^−5^	2.13 × 10^−5^	6.70 × 10^−5^	2.53 × 10^−5^	7.21 × 10^−5^	1.30 × 10^−2^	5.11 × 10^−4^
Tartrate	1.04 × 10^−5^	5.87 × 10^−5^	4.96 × 10^−7^	6.73 × 10^−5^	1.44 × 10^−5^	8.54 × 10^−5^	9.65 × 10^−6^	6.96 × 10^−5^	6.36 × 10^−5^	2.83 × 10^−4^
Aspartic acid	2.91 × 10^−6^	2.67 × 10^−5^	6.48 × 10^−7^	6.61 × 10^−5^	4.57 × 10^−6^	5.12 × 10^−5^	5.03 × 10^−6^	4.65 × 10^−5^	1.29 × 10^−5^	1.06 × 10^−4^
Glutamic acid	4.24 × 10^−7^	4.82 × 10^−5^	9.31 × 10^−7^	8.37 × 10^−5^	5.63 × 10^−6^	5.56 × 10^−5^	2.21 × 10^−5^	8.02 × 10^−5^	6.17 × 10^−4^	1.96 × 10^−4^
Lactose	3.63 × 10^−7^	5.33 × 10^−4^	9.78 × 10^−6^	2.23 × 10^−3^	2.13 × 10^−6^	1.73 × 10^−3^	2.39 × 10^−6^	1.79 × 10^−3^	2.85 × 10^−6^	3.95 × 10^−3^
Urea	9.49 × 10^−7^	1.27 × 10^−3^	1.72 × 10^−6^	2.54 × 10^−3^	2.37 × 10^−6^	1.68 × 10^−3^	2.38 × 10^−6^	1.75 × 10^−3^	1.72 × 10^−6^	2.07 × 10^−3^

## Data Availability

The original contributions presented in the study are included in the article, further inquiries can be directed to the corresponding author.
